# Salt Reduction Strategies in Portuguese School Meals, from Pre-School to Secondary Education—The Eat Mediterranean Program

**DOI:** 10.3390/nu12082213

**Published:** 2020-07-24

**Authors:** Ana Isabel Rito, Sofia Mendes, Mariana Santos, Francisco Goiana-da-Silva, Francesco Paolo Cappuccio, Stephen Whiting, Ana Dinis, Carla Rascôa, Isabel Castanheira, Ara Darzi, João Breda

**Affiliations:** 1 WHO Collaborating Centre on Nutrition and Childhood Obesity—National Institute of Health Dr. Ricardo Jorge (INSA, IP), 1649-016 Lisbon, Portugal; Mariana.Coelho@insa.min-saude.pt (M.S.); isabel.castanheira@insa.min-saude.pt (I.C.); 2 Centre for Studies and Research in Social Dynamics and Health (CEIDSS), 1649-016 Lisbon, Portugal; sofiamendes@ceidss.com; 3 National School of Public Health, NOVA University of Lisbon, 1600-560 Lisbon, Portugal; 4 Centre for Health Policy, Institute of Global Health Innovation, Imperial College London, London SW7 2AZ, UK; franciscogoianasilva@gmail.com (F.G.-d.-S.); a.darzi@imperial.ac.uk (A.D.); 5 University of Warwick, WHO Collaborating Centre for Nutrition, Warwick Medical School, Coventry CV2 2DX, UK; f.p.cappuccio@warwick.ac.uk; 6 WHO European Office for the Prevention and Control of Noncommunicable Diseases, 125009 Moscow, Russia; whitings@who.int (S.W.); rodriguesdasilvabred@who.int (J.B.); 7 Regional Health Administration of Lisbon and Tagus Valley (ARSLVT), 1700-179 Lisbon, Portugal; ana.dinis@arslvt.min-saude.pt (A.D.); carla.rascoa@arslvt.min-saude.pt (C.R.)

**Keywords:** community-based program, childhood obesity, school meals, salt intake, sodium consumption

## Abstract

High sodium (salt) consumption is associated with an increased risk of developing non-communicable diseases. However, in most European countries, Portugal included, sodium intake is still high. This study aimed to assess the sodium content of school meals before and after the Eat Mediterranean (EM) intervention—a community-based program to identify and correct nutritional deviations through the implementation of new school menus and through schools’ food handlers training. EM (2015–2017) was developed in 25 schools (pre to secondary education) of two Portuguese Municipalities, reaching students aged 3–21 years old. Samples of the complete meals (soup + main course + bread) from all schools were collected, and nutritional quality and laboratory analysis were performed to determine their nutritional composition, including sodium content. Overall, there was a significant decrease (−23%) in the mean sodium content of the complete school meals, which was mainly achieved by the significant reduction of 34% of sodium content per serving portion of soup. In conclusion, EM had a positive effect on the improvement of the school meals’ sodium content, among the participant schools. Furthermore, school setting might be ideal for nutrition literacy interventions among children, for flavors shaping, and for educating towards less salty food acceptance.

## 1. Introduction

Given the well-established evidence that excessive sodium consumption (1 g of sodium per 100 g represents 2.5 g of salt per 100 g) is linked to an increased risk of developing non-communicable diseases (NCD) [1], a reduction in population’s sodium consumption has been a key focus of both the international and national policy agendas. Reducing salt intake in the general population is not only a practical action that can prevent adverse health outcomes—such as increased blood pressure—but it is also a feasible and cost-effective strategy to reduce the growing burden of NCDs and reduce health-care costs for governments and individuals [2].

The World Health Organization (WHO) recommends a population reduction in salt intake as one of the ‘best buys’ or cost-effective actions that should be prioritized to tackle the global burden of NCDs [3,4,5]. Targets of a daily salt intake lower than 5 g for adults and 2 g for children have been recommended [6]. In addition, WHO Member States have agreed to work towards the global target of a 30% relative reduction in mean population intake of salt by 2025 relative to 2010 levels. It is crucial that this target is met in order to achieve the overall goal of a 25% reduction in premature mortality from NCDs by 2025 [2].

The overall number of countries implementing a national salt reduction strategy more than doubled from 2010 to 2015. However, despite the remarkable efforts and actions that have since been taken, more needs to be done. Data from 2013 revealed that population salt consumption in most European countries ranged from around 7 g/day (Bulgaria, Cyprus, Germany, and Latvia) to 13 g/day (Czech Republic) [7,8].

Among all dietary habits, excessive salt intake has the most adverse outcomes. The average daily intake of salt per capita among the Portuguese population is 10.7 g [9], which is double the level recommended by WHO (<5 g) [6]. Portugal ranks the highest among European countries regarding salt intake, with excessive intake reported in 63.2% of women and 88.9% of men [10]. The problem also affects younger groups, as research shows that most children and adolescents exceed daily recommendations [11,12,13].

Excessive salt intake is associated with an increased risk of obesity—partially due to poor diets that are high in both energy and salt, such as regular consumption of breakfast cereals [14] and highly processed foods [10]. Another reason for this association may be that consumption of salty foods stimulates thirst and increases fluid intake, thereby increasing the consumption of sugar-sweetened beverages, which can further fuel obesity [15]. This scenario is of particular interest in Portugal, where the prevalence of childhood overweight has been among the highest in Europe, affecting around one in three children [16]. It is, therefore, urgent to tackle this issue, as even small reductions in salt consumption can bring great health benefits to children by reducing the risk of developing cardiovascular diseases—the leading cause of death and disability in Portugal and worldwide [17,18].

The Portuguese “National Program for the Promotion of Healthy Eating” [19], in line with internationally recommended interventions [20], strongly advocates for the implementation of strategies to reduce dietary salt intake in children by providing information and education on healthy eating as well as the strengthening of consumer protections, particularly by reducing the salt content of school meals.

Several attempts have been made to reach children in schools to encourage healthier eating habits and improve the nutritional quality of the food served to them. These interventions can potentially impact all children of school age, irrespective of their ethnicity or socioeconomic group [21,22,23,24,25]. Primary and secondary schools serve at least one meal every day and can also determine the types of food and beverages that are available or served at schools (i.e., schools’ cafeterias and vending machines). Schools can positively impact eating behaviors and promote healthier eating [26,27,28], for example, by deploying nutrition education classes.

School is a key setting to deliver health education to children, promote healthy lifestyles and social equality, and to ensure access to nutritionally balanced meals, regardless of the family’s socioeconomic status [29]. In Portugal, municipalities are responsible for providing school meals (lunch) for pre-school and primary schools as well as for the management [30] of the menu. During secondary education, the supply of school meals is supported by the Directorate General of Education Institutions (DGEstE) [31], except for schools with their own cooking facilities. In Portugal, a set of guidelines for the school food supply has been established, which includes limits on the salt content of the school meal’s components—bread, soup, and the main dish [31].

Assuming that lunch represents 30% of the total energy value [32] and considering the WHO recommendation [6], 1.5 g of salt should be the maximum level in this meal. In Portugal, little is known about the nutritional composition of the complete school meal. The amount of different nutrients in food samples can be measured through laboratory analyses, using standardized techniques recommended by international organizations [33]. In the few studies that have been conducted to estimate sodium content of school meals in Portugal, mean salt content has ranged from 2.83 and 3.82 g [34,35,36], which clearly should be reduced.

Eat Mediterranean—A Program for Eliminating Dietary Inequalities in Schools (EM) [37], was a European Economic Area (EEA) Grant funded project developed as a Portuguese community-based intervention (2015 to 2017) through a multi-sectorial approach involving health, education, and political stakeholders. The program’s goal was to reduce nutritional inequalities among school-aged children through the promotion of the Mediterranean diet. The program comprised a comprehensive approach both at the individual level (child and family) and at the group/community level (nutritional education sessions at schools and improvement of school food environments). One of the objectives and key priority areas in the implementation of EM at the community level was to evaluate and improve the nutritional quality of food available in school meals. Within the school food environment, the EM program proposed a qualitative and quantitative (laboratory) analysis and evaluation of the nutritional adequacy of school meals. The aim was to identify nutritional deviations, according to international/national recommendations [29,31,38,39] and correct them by modifying the food composition of school meals through both training of the schools’ food handlers and through the development and implementation of new menus.

## 2. Materials and Methods

### 2.1. Program and Participant Schools

The EM program was implemented over two school years (2015/2016 (Y1) to 2016/2017 (Y2)) in two Portuguese municipalities: Santarém and Alpiarça. In total, 25 individual public schools and 5773 students (3–21 years old), from pre-school to secondary education, participated in EM.

The entities responsible for the supply of school meals in both pre-schools and primary schools were Santarém Municipality (17 schools) and Alpiarça Municipality (three schools). For secondary schools, DGEstE supplied meals to four schools, while one had their own cooking service.

The specific evaluation and intervention on nutritional adequacy of served school meals (lunch) were organized in three phases:

- Evaluation (Y1): 386 school menus were analyzed qualitatively. Thirty-nine school meal samples were collected from 10 kitchens that served all 25 Schools for analysis during the period between March and June 2016. A report on qualitative and quantitative nutritional adequacy of school meals was presented to school communities.
- Intervention (Y1/Y2): From July 2016 to March 2017, a working group was established to discuss the results of the report from the evaluation phase and to develop a new proposal for school menus. The working group included public health professionals, nutritionists, a municipal food engineer, school cooks, teachers, and parents. The new school menus were developed according to the WHO recommendations [29,38] and national guidelines [32,40], and these were implemented in all participant schools. Additionally, training was provided for the schools’ food handlers in order to implement the new changes. During the intervention period, nutritionists from the working group closely followed and guided every step of the process, including food preparation, cooking and serving of the meals while, at the same time, providing training to the food handlers. The training covered topics, such as food safety, cooking methods, and portion guidance (for example, to estimate the amount of salt that could be added to food, a standard measuring spoon or cup was introduced for all food handlers to use). Additionally, technical sheets of the new menus were developed, and their implementation was conducted under the supervision of members of the working group.
- Post-intervention (Y2): A new set of 39 school meal samples was collected from the same kitchens from April to June 2017, and a qualitative and quantitative evaluation of the changes was performed.

Ethical approval was granted by Lisbon and Tagus Valley Regional Health Administration Ethical Committee (089.CES/INV/2015).

### 2.2. Food Samples and Sample Preparation

Food samples were collected from all 10 kitchens that served meals to the 25 schools. Of the 10 kitchens, nine served meals at their own schools, so samples were collected at the moment of serving. One school was served by transporting meals from a central kitchen outside the city. In this case, food samples were collected at the school immediately prior to serving.

The samples consisted of the food portions that were served to children at lunchtime. Each food sample consisted of three main items: bread, soup, and the main course (including salad or cooked vegetables and one piece of fruit). These were collected during both evaluation and post-intervention phases in a total of 39 samples in each phase. In one of the schools (school B), it was not possible to analyze the bread samples, as they were not sent to the laboratory. The meal items were weighed on a Mettler-Toledo PB3002-S/FACT (Mettler-Toledo, Inc., Columbus, OH) laboratory scale, with an accuracy of 0.01 g. Samples were collected using latex gloves, placed in sterile polythene bags, and alphabetically coded to maintain confidentiality. The samples were transported to the laboratory, refrigerated, homogenized, and milled using a high-speed grinder, a knife mill Grindomix GM 200; Retsch, Haan, Germany equipped with titanium knives to prevent contamination. The prepared samples were stored in vacuum bags at the freezing temperature (−20 °C) until processing.

### 2.3. Laboratory Analysis and Interpretation

The analysis was performed in accordance with the methodology recommended by the Official Methods of Analysis of AOAC International [33], under quality assurance conditions complying with the requirements described in standard EN ISO/IEC 17025: 2005 [41]. For sodium determination, the samples were analyzed in triplicate using an inductively-coupled plasma optical emission spectrometer, ICP OES, model iCAP 6000, Thermo Fisher Scientific, Madison, WI, USA for the determination of sodium (Na) content. There are several common sources of sodium in food, including from salt added during preparation or during processing, as well as from the sodium in seasoning (e.g., sodium phosphate, sodium bicarbonate, MSG mono-glutamate, etc.). However, this study assumed that all sodium in food was in the form of sodium chloride and equivalents, so all results were expressed in terms of “salt”.

The salt content in g/100 g of food was calculated by the formula: salt (g) = sodium (g) × 2.5 [41]. Considering a school meal (lunch) makes up 30% of the daily total energy intake [32], 1.5 g of salt was the reference value used in the present study (according to the WHO recommendation of salt intake [6]: 0.30 × 5 = 1.5 g).

### 2.4. Statistical Analysis

Data sets were produced using Microsoft Excel^®^ spreadsheets, and statistical analyses were performed using IBM SPSS^®^ statistics for Windows, version 22.0, Armonk, NY, USA [42]. Results were reported as mean (+ standard deviation). Non-parametric tests for comparing means were carried out for paired samples. A significance level of α = 0.05 was considered statistically significant.

## 3. Results

The quantitative analysis of the school menus found that the standardized serving portions collected during the evaluation phase and the post-intervention phase were similar. Regarding the reduction in sodium and salt equivalent of the individual meal components, there was a 34% reduction per serving portion of soup. There were no significant changes in sodium and salt equivalent per serving portion of bread or per serving portion of the main course. In the complete meal, including the three components, there was a 23% reduction in sodium and salt equivalent per serving portion (Table 1).

**Figure 1:**
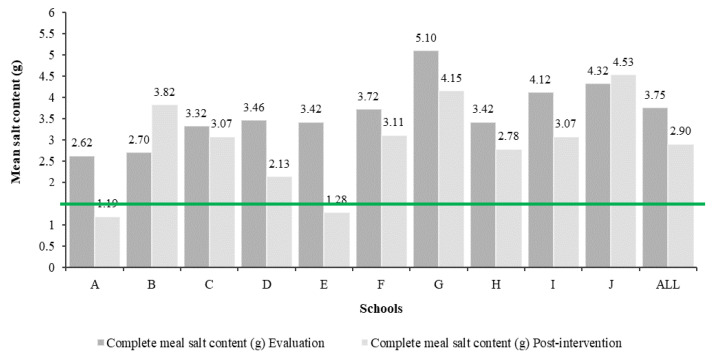
Mean salt content (g) of the complete school meals analyzed at the evaluation phase and the post-intervention phase of the eat Mediterranean program and its adequacy regarding the reference value (maximum 1.5 g of salt/meal), by the school.

Changes in the mean salt content of the complete school meal in grams (g) at evaluation and at the post-intervention phase are shown, for individual schools and for all schools combined, in Figure 1. For all schools except for two (B and J), there was a decrease in salt content between the two time-points.

## 4. Discussion

While community-based programs designed to improve the nutritional quality of school meals have been shown to be effective previously [43,44], EM was one of the first programs in Portugal to address qualitative and nutritional laboratory analysis together. Through a multidisciplinary approach targeting the school food environment, a key objective of the program was to improve the nutritional composition of school meals served to young people during lunchtime. In Portugal, addressing the quality of school meals is an important way to promote healthy diets as at least one meal is offered every day, and at pre- and primary education levels, almost every child has lunch at school [45].

The qualitative assessment of the 386 school menus has been presented elsewhere [46]. As part of evaluating EM, this study focused on identifying the nutritional deviations of sodium and salt equivalent content of school meals from international and national recommendations [29,31,38,39] and aimed to correct them by modifying the nutritional composition of school meals. This was done through training of the schools’ food handlers and the development and implementation of new menus. The results showed that EM had a positive effect on the improvement of the school meals’ salt content among the participating schools, achieving an overall reduction of 23% of the salt content of school meals served at lunchtime.

At the beginning of the EM program, the mean salt content of school meals was 3.75 g of salt per meal. These findings were similar to those reported in previous Portuguese studies [34,35,36], as well as in studies from other countries that assessed the salt content in school meals served in canteens [47]. Interventions as part of the EM program led to a significant reduction (*p* < 0.05) of salt content (from 3.75 g to 2.90 g of salt per meal, i.e., ~23%); however, it was still far from the reference value of lunch salt content (1.5 g of salt), and it was estimated that it would need to be met to achieve recommended salt consumption levels.

Looking separately at each component of the meal, the main dish was the component with the highest contribution to the salt content of the whole meal. This was also found in the study conducted by Barbosa et al. 2018 [48] in Portuguese University Canteens, in which it was suggested that one possible explanation for this result was the presence of intrinsic sodium in foods, such as meat and fish, which is higher than the sodium intrinsically present in vegetables used for soups [49]. However, we found a significant reduction in the salt content of soup from 1.48 g per serving portion before the intervention to 0.98 g per serving portion after the intervention (~34% reduction).

The values for lunch salt content, after EM intervention, are yet slightly higher than those reported by other Portuguese studies [50,51]. According to the Portuguese 2018 guidelines for menus and school canteens [31], the maximum value that can be added to soups and main dishes during the cooking process is 0.2 g of iodized salt. In addition, it is recommended that salt be replaced by glasswort or aromatic herbs. Regarding the serving of bread included in the school meals, according to the recommendations for Portuguese school meals [31], it should be one small piece of bread of 25 g for pre-school and primary school and 45 g for elementary and secondary school, with a maximum salt composition of 1%, meaning 0.25 g and 0.45 g of salt per serving of bread, respectively. As there was no intervention targeted at reducing bread provided during school meals, this study observed that, despite the level of education, the mean serving of bread was around 45 g, and the salt content of bread per serving, both before and after the intervention, was above the guidelines (0.46 g–0.49 g). In Portugal, there is a culture of always serving bread at mealtime, which is reflected in the official guidelines [31]. It could be suggested that if there was a non-mandatory offer of bread at school meals, at least for young children, and if carbohydrate intake recommendations were met through foods with less salt, a further reduction on overall salt intake could have been observed through this action alone.

There were several challenges in the implementation of the EM program. One of these was to reduce the amount of salt added by cooks while preparing the meals, as while the technical guidance clearly requires that the amount of salt added during meal preparation be accurately measured, several cooks still opted to measure by “hand”. This was also described by Gonçalves et al. [52], who found that the amount of added salt was influenced by the taste of the cook, even though many food handlers acknowledged that they did not taste the food before adding salt. That study also pointed out that food handlers were aware of the health problems associated with excessive salt intake as well as the recommended salt intake values, but they mentioned that the greatest difficulty in salt reduction was the opinion and acceptance of the consumers toward less salt in foods [52]. Such limitations reinforce the importance of educating both consumers and food handlers so that programs aiming at reducing the salt content in school meals and other settings can be more effective.

Action to reduce salt consumption is urgent, including among younger populations, in order to reduce the risk of developing cardiovascular diseases. The offer of high sodium meals in a school environment can contribute to individuals acquiring long-term poor eating habits, including increased consumption of processed food, which is already a pattern in Portuguese children [10]. Additionally, emerging evidence suggests dietary sodium intake may be associated with obesity, both through pathophysiological mechanisms and through the induction of thirst and increased consumption of high energy drinks [53,54,55,56,57], enhancing the need to address and tackle this public health issue.

The improvement of the menus introduced by the EM program has shown that it is possible to successfully reduce the salt content of school lunches through existing mechanisms. To ensure school meals are nutritionally adequate, it is essential that trained cooking staff and all responsible parties strictly comply with the technical sheets provided. It is also crucial to continue the education of students, parents, educators, teachers, as well as the monitoring of all stages of preparing and serving meals by the cooking staff. The integrated and concerted work among health departments, research institutions, municipalities, and educational communities was a strong part of the success of the EM program.

Among the common challenges educators face when trying to reduce the amount of added salt to meals is rejection by consumers due to “lack of flavor” [52]. However, the adaptive capacity of the flavors-linked neurological system to small reductions in salt in meals has been well described [57]. Thus, school settings may be ideal not only for nutrition literacy interventions among children but also for flavors, shaping and educating towards less salty food acceptance.

One of the pitfalls of this study was the limited time frame of the intervention to carry out all the activities projected in this comprehensive program without including a post-intervention longer monitoring period. This would have been important to continuously assess the adaptation to the changes implemented in the context of the new school food environment, in particular, the acceptance/preference of less salty meals by the children.

Nonetheless, recognizing the relevance of consumer acceptance in order to obtain long-lasting changes, EM paved the way for further work towards providing healthier meals in the participating schools, including a monitoring system to assess students’ acceptance of school meals changes and also regarding food waste. The training, capacity building, and nutritional education offered during the EM intervention to all school community (teachers, parents, children, food handlers, and others) would hopefully contribute to the sustainability of the progress achieved in improvements and support continuous improvements.

## 5. Conclusions

This study demonstrated the success of the EM program in reducing the salt content of lunch meals served in schools. School meals must be nutritious, and reinforcement of this through regular monitoring and evaluation is a key factor to ensure school food quality. In order for school meals to be nutritionally adequate, trained cooking staff and all responsible parties would need to strictly comply with the provided technical sheets. It is crucial that the health literacy of students, parents, educators, and teachers is developed through continuous education, and the monitoring process at all stages of preparing and serving meals by school food handlers is strengthened. This comprehensive program was built through a collaboration between different stakeholders (health departments and units, research institutions, municipalities, and educational communities), which was both key to its success and ensured a holistic approach towards promoting healthier behaviors.

## Figures and Tables

**Table 1:** Sodium and salt content of school meals components (soup, main course, and bread) and of the complete meal (all components) analyzed at the evaluation and the post-intervention phases of the eat Mediterranean program.

		Serving Portion (g) ^(^^a)^		Sodium (g/Serving Portion)			Salt (g/Serving Portion) ^(b)^		
	*n*	Evaluation	Post-Intervention	Evaluation	Post-Intervention	*p*-Value	Evaluation	Post-Intervention	*p*-Value
**Soup**	10	227.10 ± 30.24	220.30 ± 37.08	0.59 ± 0.12	0.39 ± 0.24	0.017 Change 0.20~34%	1.48 ± 0.29	0.98 ± 0.59	0.017 Change 0.49~34%
**Main course**	10	262.30 ± 51.71	269.80 ± 61.28	0.68 ± 0.21	0.60 ± 0.25	0.169	1.70 ± 0.54	1.50 ± 0.63	0.169
**Bread**	9	46.00 ± 14.80	46.56 ± 16.08	0.19 ± 0.09	0.18 ± 0.11	0.441	0.48 ± 0.21	0.45 ± 0.27	0.514
**Complete meal**	10	-	-	1.50 ± 0.30	1.16 ± 0.45	0.028 Change 0.34~23%	3.75 ±0.40	2.90 ± 1.12	0.047 Change 0.85~23%

^(a)^ There were no statistically significant differences between serving portions (g) (*p* > 0.05); ^(b)^ The salt content was calculated by the formula: salt (g) = sodium (g) × 2.5 [35].
